# High-Throughput Analysis of T-DNA Location and Structure Using Sequence Capture

**DOI:** 10.1371/journal.pone.0139672

**Published:** 2015-10-07

**Authors:** Soichi Inagaki, Isabelle M. Henry, Meric C. Lieberman, Luca Comai

**Affiliations:** 1 Plant Biology Department and Genome Center, University of California Davis, Davis, California, United States of America; 2 Department of Integrative Genetics, National Institute of Genetics, Mishima, Japan; Universidad Miguel Hernández de Elche, SPAIN

## Abstract

Agrobacterium-mediated transformation of plants with T-DNA is used both to introduce transgenes and for mutagenesis. Conventional approaches used to identify the genomic location and the structure of the inserted T-DNA are laborious and high-throughput methods using next-generation sequencing are being developed to address these problems. Here, we present a cost-effective approach that uses sequence capture targeted to the T-DNA borders to select genomic DNA fragments containing T-DNA—genome junctions, followed by Illumina sequencing to determine the location and junction structure of T-DNA insertions. Multiple probes can be mixed so that transgenic lines transformed with different T-DNA types can be processed simultaneously, using a simple, index-based pooling approach. We also developed a simple bioinformatic tool to find sequence read pairs that span the junction between the genome and T-DNA or any foreign DNA. We analyzed 29 transgenic lines of *Arabidopsis thaliana*, each containing inserts from 4 different T-DNA vectors. We determined the location of T-DNA insertions in 22 lines, 4 of which carried multiple insertion sites. Additionally, our analysis uncovered a high frequency of unconventional and complex T-DNA insertions, highlighting the needs for high-throughput methods for T-DNA localization and structural characterization. Transgene insertion events have to be fully characterized prior to use as commercial products. Our method greatly facilitates the first step of this characterization of transgenic plants by providing an efficient screen for the selection of promising lines.

## Introduction

The introduction of foreign or modified genes into plants using T-DNA transformation is a major approach used both for plant functional biology and molecular breeding purposes. Within the binary plasmid, the T-DNA can be defined by 25-base-pair border regions, which are recognized and nicked by virulence (vir) D1/D2 proteins to define a major transforming single stranded DNA species. Following entrance of the T-DNA strand in the plant nucleus, the T-DNA is integrated into the host genome via non-homologous end joining (NHEJ) repair [1]. A dsDNA intermediate may undergo end-to-end or end-to-tail multimerization or remain a single unit. The T-DNA is thought to ligate into accidental genomic dsDNA breaks via NHEJ. The result of this phase is that T-DNAs are randomly inserted into the genome, producing a variety of transformed lines each with its own specificities. Because of the random nature of the process, T-DNAs are often inserted into multiple loci or multiple copies of T-DNA are inserted into a locus [2–4]. Additionally, insertion events can encompass more than the canonical T-DNA often including some or the entirety of the plasmid backbone [4,5]. In other cases, partial copies of the T-DNA are inserted and junctions occur at sites others than the regular borders. Because transgene expression level is affected by the location of the T-DNA insertion(s), as well as their structure, e.g., copy number, inverted or tandem repeats, it is important to develop tools to rapidly characterize insertion events.

In the model plant *Arabidopsis thaliana*, T-DNAs have been used to construct sequence-indexed T-DNA insertion mutant libraries as functional genetic tool, in which each T-DNA flanking sequence tag is mapped and cataloged [6]. Conventionally, the T-DNA flanking sequence is identified using PCR-based methods, such as thermal asymmetric interlaced PCR (TAIL-PCR), adapter-ligated PCR [6], inverse PCR [7], or restriction site extension PCR (RSE-PCR) [8]. However these methods are laborious and expensive, and require dedicated facilities for high-throughput processing. Furthermore, it is difficult to identify all insertion loci and to fully characterize T-DNA structures using these methods. This is particularly problematic for lines with multi-locus or complex insertions.

With the advent of next-generation sequencing technologies, new approaches are available. For example, whole genome sequencing can be used to characterize insertions, but it is expensive, especially for species with larger genomes. Sequence capture through hybridization of specific biotinylated oligonucleotide to the target DNA can provide substantial saving by enriching for the sequences of interest. For example, the use of high-throughput Illumina sequencing following sequence capture using a biotinylated oligonucleotide corresponding to a *Mutator* (*Mu*) transposon terminal inverted repeat was successful in identifying multiple flanking sequences of *Mu* insertions in high-copy *Mu* insertion lines of maize [9]. Lapage et al [10] demonstrated the potential of this method to T-DNA characterization by combining capture with 454-sequencing. This approach, however, relied on the ~1kb length of sequence yielded by the 454 method and did not address the challenge of using shorter reads and of characterizing the multiple potential insertion modes of T-DNA. We explored further applications of sequence capture-based methods and custom-developed bioinformatic tools to determine the location of T-DNA insertions in the Arabidopsis genome. Here, we demonstrate that we can successfully identify T-DNA insertion sites in lines transformed by different vectors, using a mixture of hybridization probes targeted against the various T-DNA ends present in these vectors.

## Results

To test the suitability of sequence capture to identify the location of T-DNA insertions, we used 30 independent transgenic Arabidopsis lines generated using different binary plasmids originating from the following vectors, pPLV01 (N = 6), pPLV26 (N = 16), pCAMBIA3300 (N = 4) and BJ49 (N = 4).

### Capture sequencing

**Fig 1:**
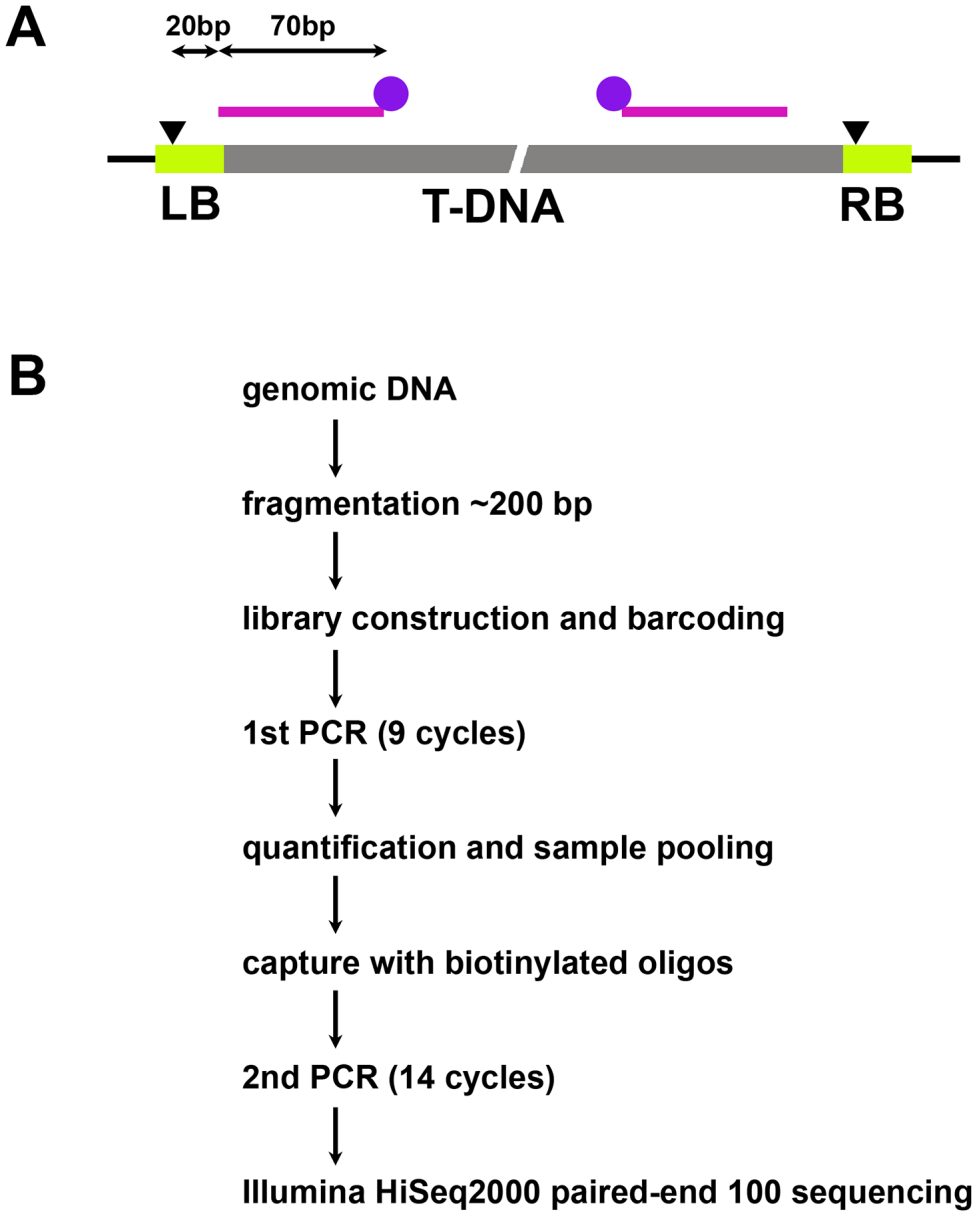
Probe design and workflow of the T-DNA capture. A. Schematic illustration of the location of the hybridization probes designed to capture T-DNA-genome junctions. The black lines represent genomic DNA. Grey rectangles represent T-DNA sequences and light green rectangles represent the left and right border repeats (LB and RB). Black triangles indicate the nicking sites. 70 mer probes represented by magenta lines are designed to match 90 bp to 20 bp (5’ to 3’) inside of the nicking sites and are 5’ biotinylated (purple circles). B. Workflow of T-DNA capture and sequencing processes.

To account for frequent deletions in the 50 bps adjacent to the T-DNA borders nicking site [11], we designed 70-mer biotinylated oligonucleotide probes that match the sequence from 90 bp to 20 bp inside of the nicking site, on both ends of the T-DNA (Fig 1A and S1 Table). Although a 25-base-pair repeat sequence within that region is shared by all vector borders, the sequence adjacent to these borders is specific to each vector. Therefore, we designed vector-specific and border-specific probes for each vector type. The sequence adjacent to the RB is shared between the pPLV01 and pPLV26 vectors, so a generic pPLV-RB probe was designed. Thus, a total of seven 5’-biotinylated probes were produced.

We extracted DNA from individual transgenic plants, and constructed Illumina sequencing libraries from each plant, using eight-base barcoded adapters (Fig 1B). After PCR amplification, approximately equal amounts of the 29 successfully prepared sequencing libraries were pooled. Library preparation for the remaining sample had failed. Next, the pooled libraries were hybridized to the cocktail of T-DNA border capture probes. After several rounds of washes, the enriched DNA fragments were amplified again and quantified. Finally enriched library DNA was sequenced for a total of ~2% of a paired-end 100 bp (PE100) Illumina HiSeq2500 lane (1/100 lane each for 2 lanes). The resulting sequencing reads were divided into individual samples based on the adapter index and filtered through several quality criteria using custom Python script (see Methods). Subsequently, 100,000 to 500,000 quality filtered read pairs were recovered from each sample (S2 Table).

### Single-end mapping of T-DNA

**Fig 2:**
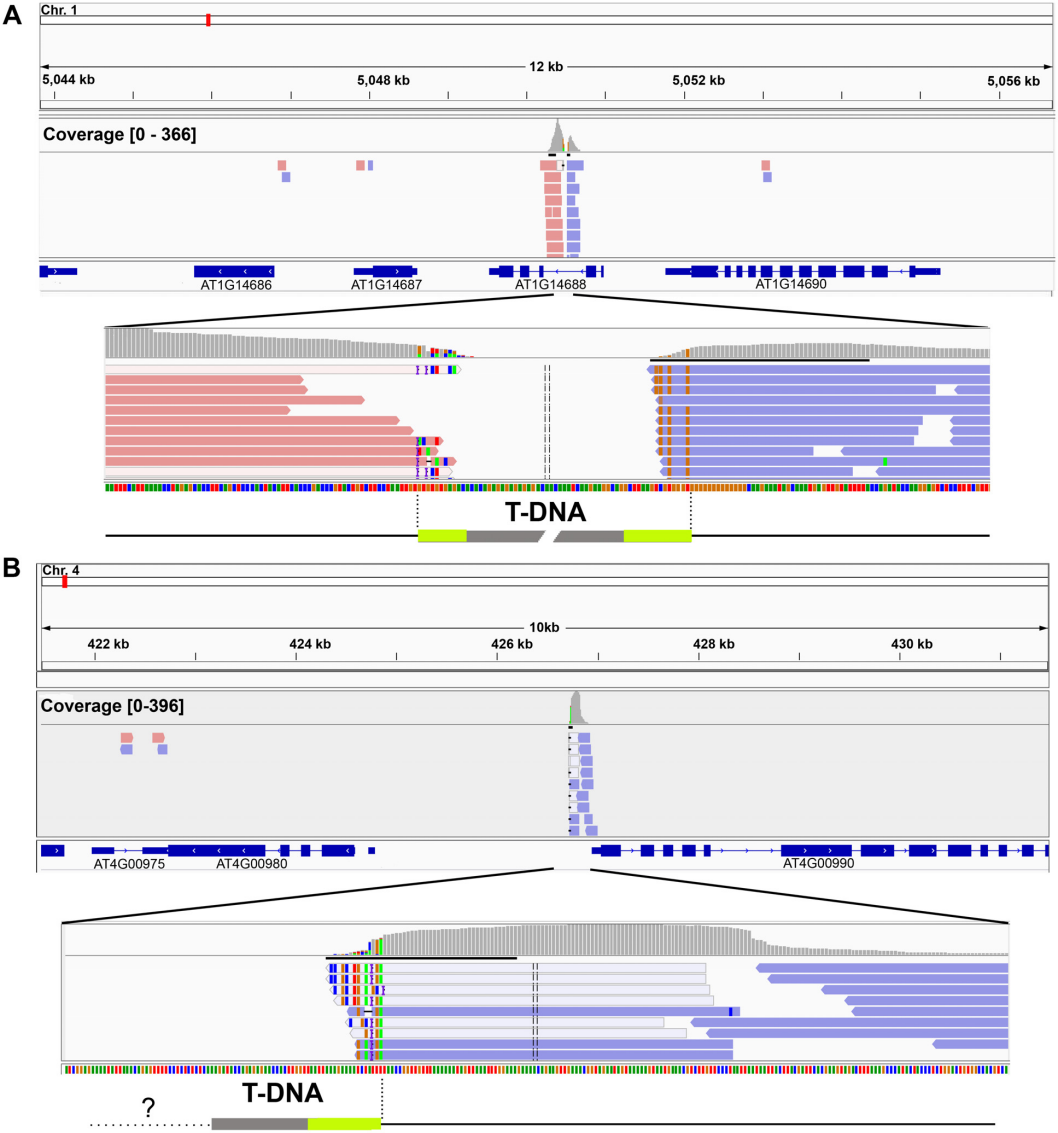
Single-end mapping of T-DNA. A, B. Genome browser view of the T-DNA insertion sites found in sample pPLV26-Cas9_C4 (A) and pPLV26-Cas9_C3 (B). Red reads map to the Watson strand and blue reads map to the Crick strand. Coverage distribution tracks are positioned above (grey histograms). Schematic representations of the insert-T-DNA junctions are represented below (T-DNA in grey, border sequences in light green and genomic sequences in black). For pPLV26-Cas9_C3, the peak shown was the only one detected and only one end of the insertion was recovered.

Because single-end sequencing is more economical than paired-end sequencing, we first tested if we could identify T-DNA insertion locations using single-ended sequencing reads. Each read pair was used as two single-ended reads and all reads were aligned onto the *Arabidopsis thaliana* genomic reference TAIR10 using Bowtie2 [12] and single-end alignment mode with default parameters. The resulting SAM files, in which each read is associated to its genomic mapping coordinates, were converted to BAM files, a binary encoding form of SAM, and sorted using SAMtools [13]. Sorted BAM files were then used to identify peaks of reads using the Model-based Analysis of ChIP-Seq version 2 (MACS2) program [14]. MACS was designed to detect peaks in ChIP-seq experiments. It compares the distribution of DNA reads from immunoprecipitated (IP) chromatin and input control reads to find peaks of sequence coverage that correspond to chromatin enriched for the target epitope. To identify peaks of reads corresponding to T-DNA insertions that are specific to a sample of interest, we input the BAM file from a given sample as “IP”, and the BAM file from another sample transformed with same construct and in same background as “input”. Specific peaks with high statistical significance were manually inspected on the Integrative Genome Viewer (IGV) to confirm that they are distinct peaks and specific to the sample of interest. We explored 29 samples carrying T-DNA inserts from four different vectors and found specific peaks from 17 samples, of which two exhibited specific peaks at three different sites and another two at two sites. Of 23 insertion sites from 17 samples, 10 sites exhibited symmetrical peaks, i.e. displayed read sets with the same orientation flanking a central genomic position, indicating that both ends of the T-DNA insertion were recovered and associated to the same locus (Fig 2A and S2 Table). In contrast, the other 13 sites showed asymmetrical peaks (Fig 2B and S2 Table), which suggests that either only one flank of the T-DNA insertion was recovered by the capture or that the T-DNA insertion was likely to involve a genomic rearrangement. No specific peak could be identified for the remaining 12 samples.

Chimeric reads that contain both *A*. *thaliana* genomic and T-DNA sequences are difficult to map. To reduce the chance of chimeric mapping and maximize the number of reads that can map to T-DNA insertion sites, we next restricted our analysis to the first 50 bases of each 100 bases. Because most of the reads are 100-base-long, we used the -3/—trim3 Bowtie 2 option to trim 50 bases from the 3’ end of each read. As a result, we identified specific peaks indicating T-DNA insertion sites in an additional 3 samples, of which one peak was symmetrical and the other two were asymmetrical (S2 Table). Overall, we identified 26 T-DNA insertion sites from 20 / 29 samples by single-end mapping.

### Paired-end mapping of T-DNA

**Fig 3:**
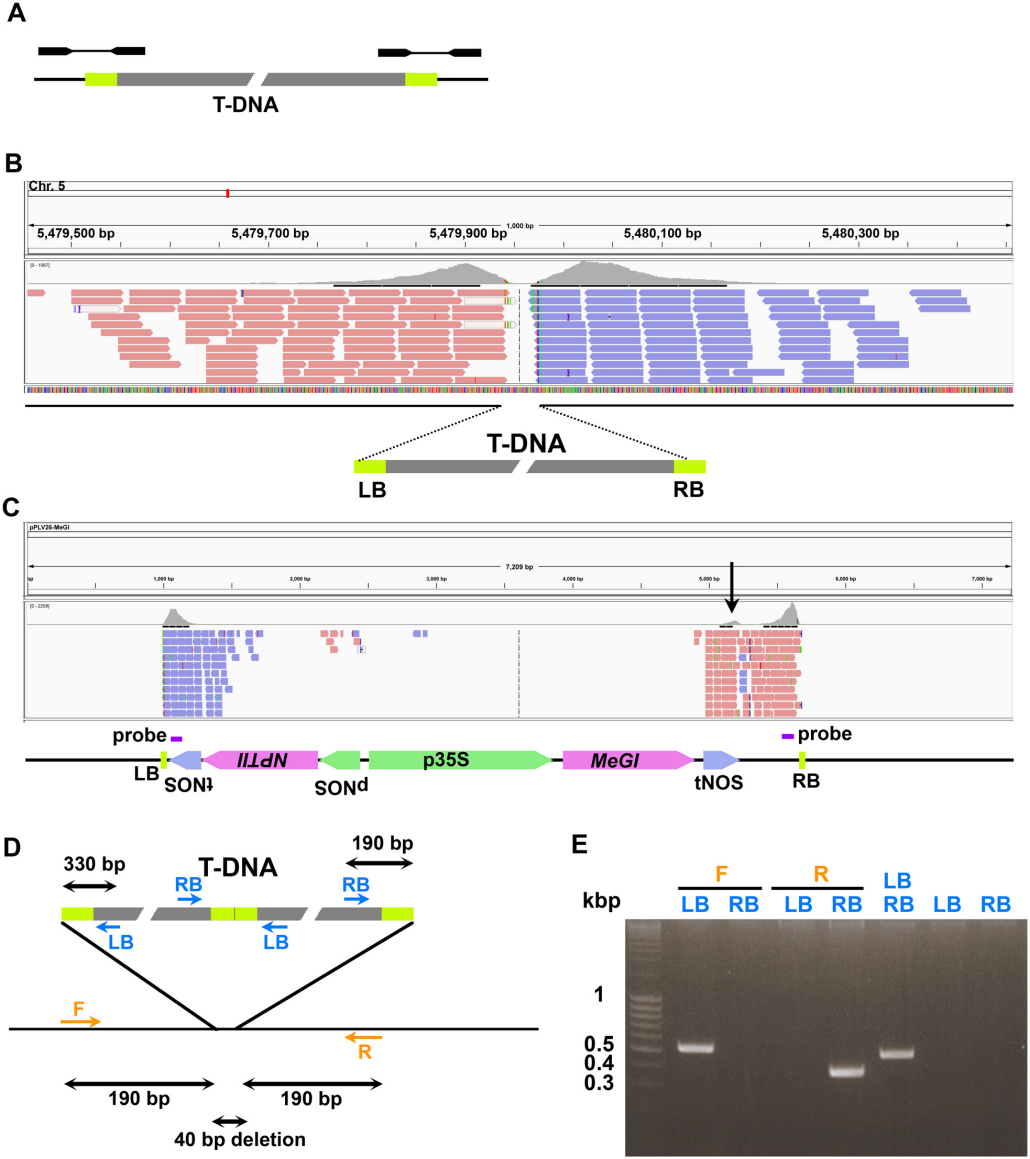
Paired-end mapping of T-DNA. A. Schematic view of read pairs that span the T-DNA—genome junctions. One read maps to the end of the T-DNA and the paired read maps to the genomic sequence around the T-DNA insertion site. B. Genome browser view showing junction reads mapping to the genomic DNA flanking the T-DNA insertion site in sample pPLV26-MeGI_2–3. C. Genome browser view of aligned reads from the same junction read pairs showing the end of the reads that are mapping to the T-DNA plasmid sequences. Elements in the T-DNA plasmid and the locations of probes for capture are shown below. The extra peak in tNOS downstream of the *MeGI* gene (black arrow) is due to the presence of another tNOS adjacent to the LB and the fact that Bowtie2 randomly selects among the best alignments when more than one is present. D. Schematic illustration of the inferred structure of the T-DNA insertion in sample pPLV26-MeGI_2–3 and primers that were used to confirm the insertion site. E. PCR confirmation of the T-DNA insertion structure in pPLV26-MeGI_2–3.

Next, we explored an approach that utilizes paired-end information to identify T-DNA locations and the precise structure of the T-DNA—genome junctions (Fig 3A). For each vector type, the reference *Arabidopsis thaliana* genome TAIR10 and the sequence of the T-DNA plasmids used were combined *in silico* to create a new “genome & T-DNA” reference genome. Paired-end reads were aligned onto this reference using Bowtie2 in single-end alignment mode with -3 50 option. We reasoned that single-end alignment is more efficient in aligning “discordant pair”, where mates map to distant location of a chromosome or different chromosomes because each read is independently aligned. After single-end alignment, read pair information was used to look for sequence junctions.

After alignment, we screened the reads for pairs in which one mate mapped to the T-DNA sequence and the other to genomic sequence, using a custom Python script (see Methods). We identified 28 sample-specific T-DNA—genome junctions in 22 / 29 samples (Fig 3B and S2 Table). Of these, 26 were consistent with peaks previously identified using single-end mapping while insertion sites were newly identified from two samples (S2 Table). Of those 22 samples, two samples exhibited three specific junctions and two exhibited two specific junctions, consistent with the result of the single-end analysis. In a total of 28 insertion peaks, 11 were symmetrical. No clear peak of junction reads could be identified for the remaining 7 samples.

### Assessment of T-DNA structure

Next, we investigated the structure of the T-DNA insertions. We used paired-end information to determine which T-DNA border (or other sequence from the T-DNA plasmid) flanks the genomic DNA (Fig 3C and S2 Table). In most of cases, T-DNA border sequences or nearby sequences form the junction, but in two cases, other plasmid sequences flank genomic sequences. In one of these lines (pPLV26-Cas9_C2), T-DNA internal sequences located near the Nopaline synthase promoter (pNOS), upstream of the kanamycin resistance gene NPTII, defined both ends of the T-DNA insertion. This type of junction is not expected to be detected using this method since our capture probes targeted the T-DNA border regions only. However, since we used a mixture of probes in order to capture sequences from a variety of different vectors (see Methods), it is possible that the capture probes intended to hybridize to a T-DNAs cross-hybridized with internal sequences (e.g., cloning sites, common primer binding sites) from another T-DNA plasmid. Indeed, 47 nucleotides in the 5’ region of the BJ49-RB probe perfectly match the sequence of pPLV26-Cas9 that correspond to the location of the peak detected in pPLV26-Cas9_C2. In this sample, another junction between a sequence of the CaMV 35S promoter and the RB sequence was detected, which was probably captured by the pPLV-RB probe, indicating that the structure of T-DNA insertion is likely to be complex. Similarly, an undetermined cross-hybridization may have played a role in capturing the junction between vector backbone sequence and genomic DNA in sample pPLV01-AtU6:gRNA_C3 (S2 Table).

Insertion of vector backbone is very common in agrobacterium-mediated transformation [4]. Thus we next screened our data for the presence of reads mapping to the vector backbone. We found evidence of backbone insertion in 16/29 samples (S2 Table). This is consistent with previous result showing that about half of transgenic lines carry vector backbone insertion [4].

### Result validation

To confirm the location of the T-DNA insertions and their junction structure, primers were designed for 7 samples for which a single T-DNA insertion site had been identified. LB and RB primers were designed near the T-DNA borders and directed outwards, while the F and R primers were designed on the Arabidopsis genome facing toward the T-DNA insertions site, to amplify fragments spanning the T-DNA—genome junctions (Fig 3D and S1 Table). Consistent with the sequencing results, we could amplify each specific fragment (Fig 3E and S1 Fig). Following Sanger sequencing of the PCR products we confirmed the structure of the T-DNA—genome junctions. The T-DNA insertion site from one of the samples for which very few junction reads were obtained (pCAMBIA3300-pFWA:H2B-CFP_1; S2 Table) was confirmed by PCR as well (S1 Fig). These results validated the use of capture sequencing and analysis to identify T-DNA location and determine the basic structure of the T-DNA insertion sites.

## Discussion

Here we describe an efficient and low-cost method (S3 Table) for T-DNA mapping using sequence capture followed by short read sequencing. Similar methods for mapping of T-DNA or (retro)transposon insertions using next-generation sequencing were recently reported [9,10,15]. These reports successfully identified insertion sites of T-DNA/(retro)transposon from various species (Arabidopsis, maize, and a legume *Lotus japonicus*) using different combinations of target enrichment methods (capture or PCR-based method), pooling method (multi-dimensional pooling and index-based pooling) and different high-throughput sequencing technologies (Illumina short read or Roche 454 long read).

Here we propose a simple method, well suited to the analysis of small or large sample number. We elected to employ sequence capture rather than a PCR-based method because probes from different T-DNA plasmids could be mixed so that the hybridization of probes to samples carrying T-DNA from different origins could be carried out in a single tube. This feature of our method is advantageous for researches working with multiple transgene constructs. Using only 2 probes per T-DNA (LB and RB), we were able to recover insertion sites in 75% of the samples (22/29), suggesting that capture with a minimum probe set is a good choice, particularly when working with multiple transgene constructs. Multi-dimensional pooling of samples is cost-effective when working with a large number of samples [10], but for relatively small number of samples (up to 100 samples), index-based pooling provides an easy and rapid way of identifying T-DNA flanking sequences and it obviates the need for further deconvolution of mixed samples by PCR of 2D-pools [10].

Our results suggest that short read sequencing (i.e. truncating the reads at 50b) is efficient for T-DNA mapping and initial determination of junction structure. Paired-end reads were more efficient for T-DNA mapping than single-end reads and are required to determine junction structure. However, junction structure could be determined with PCR after single-end sequencing and mapping. Thus, depending on the situation both single-end and paired-end sequencing can be appropriate. Our results suggest that a small fraction of a HiSeq lane (~5.8M reads) is sufficient to map the T-DNA insertions in ~20 samples. Therefore, by mixing indexed libraries with other sequencing samples, T-DNA mapping can be carried out cost-effectively. Taken together, our results suggest that sequence capture targeted against border sequences only, followed by indexed-based pooling and paired-end short read sequencing, combined with our rapid and simple bioinformatics pipeline provides a cost-effective method for the localization and structural characterization of T-DNA insertions. Compared to other methods, this method is not as thorough because it is not designed to detect non-canonical events, i.e., insertions that do not end with the T-DNA borders. On the other hands, this method is significantly cheaper and does provide a very rapid tool to screen high number of lines for those that are consistent with single, conventional insertions events. More thorough analyses can follow on a much reduced number of lines subsequently.

We were not able to detect insertion sites in 7 out of 29 samples analyzed. Additionally, for many insertion sites, only one end of T-DNA—chromosome junction could be recovered (17/28). However, depending on the sample, 3% to 60% of read pairs mapped to T-DNA or T-DNA—chromosome junction (S2 Table), suggesting that the sequence capture process was successful at recovering specific fragments in all samples. Indeed, analysis of the distribution of the reads that mapped onto the T-DNA sequence shows that most of the captured reads mapped to regions surrounding the borders even in samples for which a specific junction could not be identified. In addition, all of the samples for which no T-DNA—genome junction could be identified contained vector backbone insertions (S2 Table), suggesting that “pass through” of borders occurs frequently and that the inserted T-DNA is not terminated at canonical T-DNA borders in these samples. This is consistent with the idea that those samples carry unconventional junctions, which were indeed detected in 3 samples. To detect all junctions including unconventional ones, a capture using probes that tile the entire sequence of the T-DNA plasmid would be preferable. This is possible but when the goal is to screen transgenic lines for conventional border-to-border insertions, our approach using exclusively border probes is the most cost-effective.

In conclusion, we developed a rapid and cost-effective method for the identification of T-DNA flanking sequences and the structural characterization of T-DNA insertion. This approach is applicable to any research involving transgenic organisms, either for basic biology or for molecular breeding. Our results confirmed the presence of frequent rearrangements within the inserted T-DNA sequences, including unconventional junctions and backbone insertions. In our dataset, only 3 samples exhibited clear LB to RB insertion, of which two carried insertions at multiple sites. Rearranged and complex T-DNA insertions may affect the expression and the function of transgenes and complicate regulatory processes for transgenic events targeted for agricultural use. Efficient ways of characterizing T-DNA insertions in order to screen for lines with “clean” insertions are needed. The bioinformatic tools developed here to search for T-DNA—chromosome junctions are simple and can be applied for finding flanking sequence of transposons or any foreign DNA.

## Materials and Methods

### Plant Materials and Growth Condition

*Arabidopsis thaliana* background Columbia–0 (Col–0) or Landsberg *erecta* (L*er*) were transformed using the standard floral dip method [16] using *Agrobacterium tumefaciens* strain GV3101::pMP90 [17]. Plants were grown in Sunshine Professional Peat-Lite Mix 4 (SunGro Horticulture) in a controlled environment growth room at 20°±3° with a 16 h/8 h light/dark photoperiod. Transformed plants were selected on solid Murashige and Skoog (MS) media containing 40 mg/l kanamycin (pPLV26) or 15 mg/l hygromycin (BJ49) or on soil with BASTA spray (final concentration of glufosinate-ammonium, 0.00578%; pCAMBIA3300, pPLV01). In the T2 generation (the selfed progeny of primary transoformants), the expected 3:1 segregation ratio for single-locus insertion was verified with antibiotics or herbicide resistance except for plants transformed with BJ49-35S:Cas9-GR and pPLV26-MeGI, which were not analyzed for segregation.

### T-DNA plasmids

pPLV26-Cas9 and pPLV01-AtU6:gRNA were constructed using the pPLV26 and pPLV01 vectors, respectively [18]. pPLV26-*MeGI* was characterized previously [19]. BJ49-35S:Cas9-GR was constructed using the BJ49 binary vector [20]. pCAMBIA3300-pFWA:HTB2-CFP was constructed using a modified pCAMBIA3300 (CAMBIA) and transformed into GFP-*tailswap cenh3-1* plants [21]. The detailed description of the plasmid constructs used in this study is beyond the scope of this publication. However, those plasmids and the sequences are available upon request.

### Preparation of Capture Libraries

Genomic DNA from each transgenic plant was isolated using the Genomic DNA Mini Kit for Plant (Geneaid). DNA was quantified using a Qubit fluorometer (Life Technologies). 500 ng of genomic DNA from each sample was fragmented using 1μl of double-stranded DNA Fragmentase (New England Biolabs) for 15 minutes to 45 minutes to yield roughly 100 bp to 500 bp fragments. After purifying DNA fragments with AMPure beads (Beckman Coulter) with a sample to AMPure ratio of 1 to 1.8, Illumina sequencing libraries were prepared using the KAPA HTP Library Preparation Kit Illumina platforms (Kapa Biosystems), and custom synthesized eight bp barcoded adapters (S2 Table) following the manufacturer’s protocol. After adapter ligation, libraries were selected for fragment size ranging from 250 bp to 450 bp using AMPure beads according to the protocol of KAPA HTP Library Preparation Kit. The pre-capture amplification step included 9 cycles of amplification using the KAPA HiFi HotStart ReadyMix (Kapa Biosystems) and following the standard NimbleGen protocol. The resulting libraries were checked for insert size using agarose gel electrophoresis and quantified using a Qubit fluorometer. Forty ng of each library DNA were pooled together prior to hybridization with the capture oligonucleotides.

Hybridization was performed using NimbleGen SeqCap EZ Hybridization and Wash Kit (Roche) following NimbleGen’s protocol and [22]. The pooled library DNA was hybridized to a mixture of 5’-biotinylated 70-mer oligonucleotide probes corresponding to the T-DNA ends (Life Technologies), collected using Streptavidin magnetic beads, washed, and amplified using KAPA HiFi HotStart ReadyMix for 14 cycles. The insert size and quality of the captured library was checked with agarose electrophoresis, quantified using a Qubit fluorometer and sequenced on an Illumina HiSeq 2500 to obtain 100 bp paired-end reads, as well as 8 bp indexed reads. The pooled capture library was mixed with other unrelated barcoded samples such that it accounted for approximately 1% of the total pooled library mix. The pool was sequenced in two lanes. A list of reagents used and corresponding costs can be found in S3 Table. All sequence data have been deposited in the SRA database under BioProject ID PRJNA287142 and SRA ID: SRP059868.

### Data analysis

Sequencing reads were divided by sample based on the sequenced index reads, with one mismatch allowed. At the same time, reads were trimmed for quality (minimum mean PHRED score of 20 over a 5 bp sliding window), and reads containing adapter sequences or N bases or reads that were shorter than 35 bp after trimming were discarded. These processes were done using a custom Python script (available at http://comailab.genomecenter.ucdavis.edu/index.php/Barcoded_data_preparation_tools). The numbers of read pairs obtained for each capture library are indicated in S2 Table. In total, approximately 5.8 million paired-ended reads were obtained.

For “single-end mapping”, quality-filtered reads were aligned to the TAIR10 reference sequence of *Arabidopsis thaliana* using Bowtie2 [12] in single-end alignment mode with default parameters (—sensitive), with or without the -3 50 option (see Result section). The resulting SAM files were converted to BAM files, and sorted using SAMtools [13]. Sorted BAM files were then used to identify peaks of reads using the MACS2 program [14] with—nomodel option.

For “paired-end mapping”, quality-filtered reads were aligned to a in silico-assembled reference genome containing the TAIR10 reference sequence of *Arabidopsis thaliana* and the sequence of the T-DNA used to transform the corresponding plants, using Bowtie2 in single-end alignment mode with -3 50 option, which trims 50 bases from the 3’ end of each read. The resulting SAM files were used to find junction read pairs, for which one read maps to the T-DNA sequence and the other maps to one of the *A*. *thaliana* chromosomes, using custom Python scripts (Script 1 and 2 in S1 Text). Peaks of junction reads were identified by binning junction reads into consecutive non-overlapping 1-kb windows across each of the *A*. *thaliana* chromosomes and T-DNA and identified windows with high junction read numbers, using a custom Python script (Script 3 in S1 Text). Importantly, all samples that had been transformed with the same T-DNA vector were analyzed simultaneously such that junction read coverage could be compared between individuals for each bin. Candidate junctions were discarded if they were present in more than one individual transformed with the same T-DNA plasmid, with the rationale that insertion events are expected to be random.

## Supporting Information

S1 Fig PCR confirmation of the location of the T-DNA insertion. Details about the location of the primers used for the PCR amplification of the T-DNA—genome junctions is the same as in Fig 3D.
(TIF)

S1 Table List of oligonucleotide probes and primers. (XLSX)

S2 Table Summary of the T-DNA capture results. (XLSX)

S3 Table Cost of capture libraries production and sequencing. (XLSX)

S1 Text Python scripts. (TXT)
