# Supplementary figures and images for: High-Throughput Analysis of T-DNA Location and Structure Using Sequence Capture
Source: PLoS One. 2015 Oct 7;10(10):e0139672. doi: 10.1371/journal.pone.0139672 (PMC4596565; doi:10.1371/journal.pone.0139672)

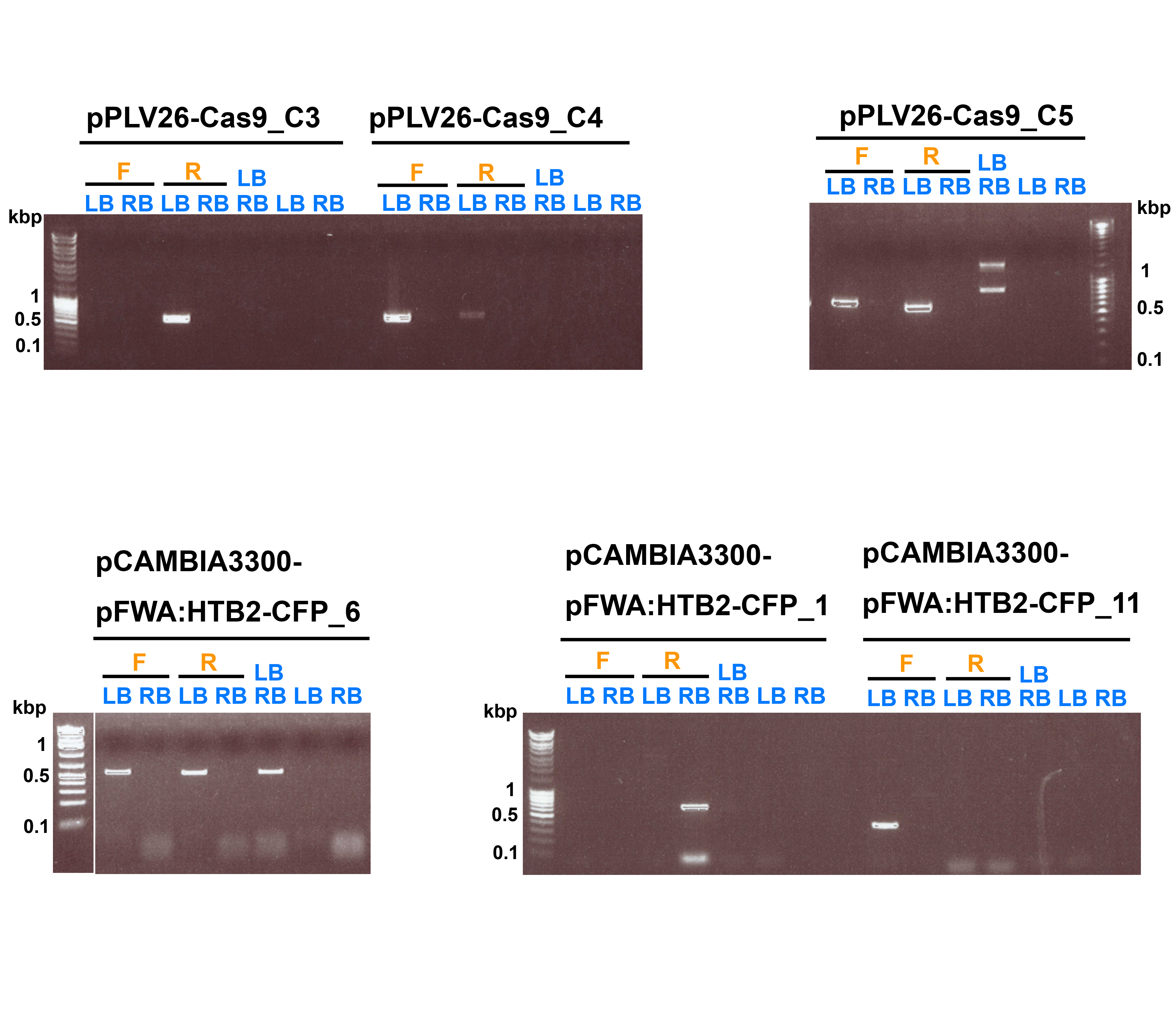

Supplement: S1 Fig — Details about the location of the primers used for the PCR amplification of the T-DNA—genome junctions is the same as in Fig 3D. (TIF) [file pone.0139672.s001.tif]
